# Eye Habits Affect the Prevalence of Asthenopia in Patients with Myopia

**DOI:** 10.1155/2022/8669217

**Published:** 2022-10-17

**Authors:** Jing Wang, Peng Zeng, Xiao-wen Deng, Jia-qi Liang, Yun-ru Liao, Shu-xian Fan, Jian-hui Xiao

**Affiliations:** Department of Ophthalmology, Sun Yat-sen Memorial Hospital, Sun Yat-sen University, Guangzhou 510120, China

## Abstract

**Purpose:**

This study aims to explore the risk factors of asthenopia in the myopic population.

**Methods:**

In this cross-sectional study, myopia patients were inquired about their eye habits and were requested to complete an asthenopia questionnaire and ocular examinations. Age, gender, occupation, anisometropia, eye care education, weekly outdoor activity time, duration of continuous near work, daily screen time, dry eye, near phoria, and binocular accommodative facility were calculated using the Student's test-test, Mann Whitney *U* test, and Pearson's chi-square test. Spherical equivalents and astigmatism were calculated using a generalized estimating equation. Binary logistic regression was performed on factors with a *p*-value <0.05.

**Results:**

Of the 65 myopic patients, 57% showed asthenopia, 52% experienced blurry vision, 37% felt their eyes hurt or sore, and 28% felt tired when performing close work. Asthenopia patients were older than patients without asthenopia (*Z* = −2.887, *p*=0.004). Daily screen time, continuous near-work time, eye care education, and dry eye were positively correlated with asthenopia (*χ*^2^ = 8.64, *p*=0.003; *χ*2 = 13.873, *p* < 0.001, *χ*2 = 9.643, *p*=0.002; *χ*2 = 7.035, *p*=0.008). After eliminating collinearity, eye care education and continuous near-work time were identified as independent risk factors of asthenopia, with odds ratios of 0.115 and 4.227, respectively.

**Conclusion:**

This study shows that receiving eye care education from schools and hospitals and limiting near-work duration to less than 45 minutes per session could reduce the occurrence of asthenopia in myopic patients. This approach may be a more economical and convenient way for myopic people to relieve asthenopia.

## 1. Introduction

Asthenopia, also known as visual fatigue, is a group of syndromes caused by various causes due to the excessive use of the eye. A group of symptoms, such as abnormal visual function, ocular discomfort, and systemic symptoms, affect normal visual function after using the eye excessively [1–4]. The clinical symptoms of asthenopia vary, including temporary blurred or double vision, eye pain and soreness, dry eyes, easy fatigue, headache, and memory loss [5]. With the development of modern civilization and the popularity of video terminals, the incidence of asthenopia has increased year by year [6]. Epidemiological studies show that 23% of school-age children [7], 64%–90% of computer users [8–10], and 71.3% of dry eye patients have varying degrees of asthenopia symptoms [11]. In addition, astigmatism, accommodation and convergence dysfunction, inappropriate lighting, and the use of video terminals are all risk factors for asthenopia [12–14]. Previous studies have shown that uncorrected refractive errors are associated with the incidence of asthenopia.

Refractive errors are responsible for one-third of blindness cases worldwide, mainly in the form of myopia [15]. Myopia, defined as spherical equivalent (SE) ≤ −0.5D, currently affects nearly 30% of the world's population. The prevalence of myopia is increasing yearly, and by 2050, myopia is predicted to affect approximately 50% of the global population [16]. Myopia can be corrected by glasses, contact lenses, and surgery, such as photorefractive keratectomy [17, 18]. The prevalence of myopia in East and Southeast Asia, including China, remains high, which brings many social and personal burdens [19–22]. Myopia causes many visual symptoms, including blurred vision, double vision, diplopia, dry eyes, eye pain, and asthenopia; all these symptoms affect people's studies, work, and quality of life [23, 24].

In the past, research on asthenopia was mainly investigated school-age children, adolescents, college students, and people with frequent use of video terminals [1, 5, 8]. Reports on the risk factors for asthenopia in people with myopia are limited.

Accordingly, this study aimed to investigate the risk factors for asthenopia in people with myopia through a cross-sectional study. The research content mainly included the symptoms and frequency of asthenopia, eye habits, eye care education and its location, ocular diopter, near phoria, accommodation facility, and dry eyes. By exploring the possible risk factors for asthenopia in the myopic population, we hope to help myopic patients eliminate the problems caused by asthenopia.

## 2. Patients and Methods

The study was conducted at Sun Yat-sen Memorial Hospital, Sun Yat-sen University, Guangzhou, China, from January 2021 to March 2021. The Sun Yat-sen Memorial Hospital Ethical Committee approved the study protocol according to the Declaration of Helsinki. Informed consent was obtained from all participants. The participants included myopic patients aged 9–40 years who could cooperate to complete the questionnaire on asthenopia. The exclusion criteria included unwillingness to participate in the study; lesions of the ocular anterior segment and fundus, such as severe dry eyes complicated by a corneal epithelial defect; history of ocular trauma and intraocular surgery; systemic diseases affecting binocular symptoms or vision, such as Sjögren's syndrome, anxiety, hypertension, and diabetes; the use of ocular or systemic medications; strabismus; amblyopia; and best-corrected visual acuity (BCVA) < 20/25 in either eye.

According to the definition of asthenopia in China's 2014 expert consensus for the diagnosis and treatment of asthenopia, we adopted an asthenopia questionnaire as shown in Annexure 1 [14, 25]. Each participant was asked the frequency of asthenopia symptoms: 0, 1, 2, 3, or 4 for never, infrequently, sometimes, fairly often, or always, respectively. A total score ≥3 was considered asthenopia in this research, suggesting that at least one symptom occurs frequently or at least 2 symptoms were present. Sixty-five patients completed the asthenopia questionnaire. The collected data included gender, age, occupation, eye care education, weekly outdoor activity time, continuous near-work duration, and daily screen time. Near work includes activities performed at a short working distance, such as reading, studying (doing homework or writing), or computer use (playing video games) [26, 27].

Ocular examinations included subjective refraction, BCVA, slit-lamp, fundus examination, sodium fluorescein staining examination, near phoria, cover-test, and binocular accommodative facility. All 65 participants completed the subjective refraction and sodium fluorescein staining examination, and participants under 18 years of age received mydriatic refraction with compound tropicamide eye drops. Anisometropia was defined as a binocular SE difference ≥1.0D between the eyes [1]. According to China's 2013 expert consensus for the diagnosis and treatment of dry eyes, any one of the symptoms of dry eyes, including burning sensation and foreign body sensation, accompanied by break-up time (BUT) ≤5 s, can be diagnosed as dry eye. Patients with a positive corneal fluorescein staining examination were excluded. A cover test was used to test the patients' near phoria, which was recorded as near exophoria and no near exophoria. The binocular accommodative facility was measured using the flipper bar method with ±2.00 D accommodative flippers and 20/30 vision cards. When the −2D and +2D lenses are flipped to see the card clearly, it is recorded as 1 cpm. Thirty-three participants completed the examination of nearby exophoria and accommodative facilities.

### 2.1. Statistical Analysis

Statistical analyses were performed using SPSS (Statistical Package for Social Sciences; SPSS Inc. IBM, Armonk, NY) version 25.0. Data are expressed as the means ± standard deviation, M (P25, P75) and *n* (%). A *t*-test or Mann-Whitney *U* test was used for continuous variables, including binocular accommodative facility and age. Categorical variables were calculated using Pearson's chi-square test or Fisher's exact test. SE and astigmatism were calculated by a generalized estimating equation (GEE). The Bonferroni adjusted significance level of 0.017 (0.05/3) was applied to eye care education, weekly outdoor activity time, and daily screen time; *p*-value <0.017 was statistically significant. For other data, *p*-value <0.05 was statistically significant. Factors that were statistically significant in univariate analyses were selected as candidate variates for multivariate analysis using binary logistic regression.

## 3. Results

**Figure 1:**
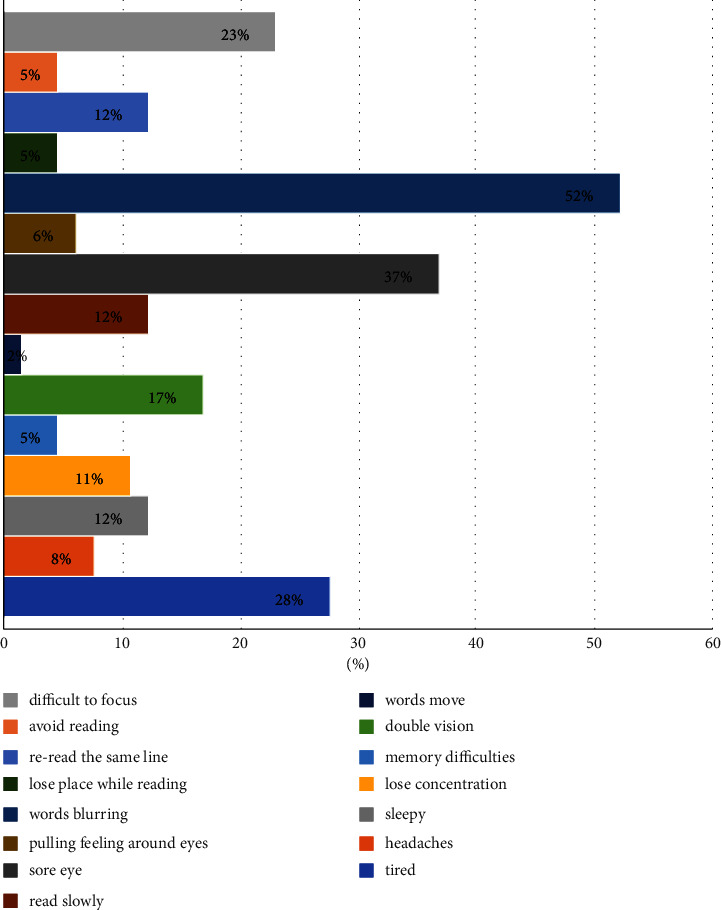
The prevalence of asthenopia symptoms.

Sixty-five patients with myopia participated in this research. By analyzing the questionnaire responses, we found that more than half of the participants (52%) experienced blurry vision when they read or performed close work, 37% felt their eyes hurt or sore, and 28% felt tired.  Figure 1 illustrates the prevalence of asthenopia symptoms.

The prevalence of asthenopia symptoms is shown in Figure 1. The most common symptoms are feeling words blurring, eyes hurting or sore, and feeling tired when patients with myopia read or do close work.

As shown in Table 1, 37 of the 65 myopic patients suffered from asthenopia, resulting in a prevalence rate of 57%. The median age of the asthenopia patients was 20 years old, which was higher than the 14.5 years old in participants without asthenopia, *Z* = −2.887, *p*=0.004. Thirteen of 15 dry eye patients had asthenopia (87%), which was higher than the prevalence rate of asthenopia in patients without dry eyes (48%), *χ*2 = 7.035, *p*=0.008.

Our study showed that the prevalence of asthenopia was 40% and 64% when myopic patients spent more than 5 hours and 2–5 hours per week on outdoor activity, respectively (Fisher, *p*=1.0). Patients who spent less than 2 hours on outdoor activity had an asthenopia prevalence rate of 69% (*χ*2 = 4.398, *p*=0.036 > 0.017). No significant difference was noted between these 3 groups. In our study, myopic patients with more than 8 hours of daily screen time had a higher incidence of asthenopia than patients with less than 5 hours (*χ*^2^ = 8.64, *p*=0.003 < 0.017). No significant difference in the incidence of asthenopia was noted compared with the 5–8 hour daily screen time group (Fisher, *p*=0.701). Among the 44 myopic patients whose continuous near-work time was more than 45 minutes, 32 (73%) had asthenopia. This result was higher than the 5 asthenopia patients of the 21 participants (24%) whose continuous near-work time was less than 45 minutes, *χ*^2^ = 13.873, *p* < 0.001 (Table 1).

Among the 40 participants who received eye care education in schools or hospitals, 15 (38%) had asthenopia, which was lower than the prevalence rate of 12 people (86%) who had not received education (*χ*^2^ = 9.643, *p*=0.002 < 0.017) and 10 people (90%) who said they received eye care education from the media (*χ*^2^ = 9.848, *p*=0.002). The prevalence rate of asthenopia between media and not receiving eye care education had no significant statistical difference (Fisher, *p*=1.0). No statistically significant differences were noted in gender, occupation, diopter, astigmatism, anisometropia, binocular accommodative facility, or near exophoria between asthenopia and non-asthenopia myopic patients, as shown in Tables 1 and 2.

(See Table 3) presents the logistic regression model results indicating that eye care education and continuous near-work time were independent associated risk factors of asthenopia, with odds ratios of 0.115 and 4.227, respectively.

## 4. Discussion

The prevalence of myopia in China remains high. Past research showed that 78.4% of Chinese middle school students and 90% of Chinese teenagers and young adults have myopia [21, 28]. People with myopia are often accompanied by asthenopia. Asthenopia may cause many uncomfortable symptoms, including nonpersistent near vision, blurred vision, difficulty focusing, and physical symptoms, such as memory loss and headaches; all these symptoms seriously affect people's studies and work [3, 7]. Our study aims to help myopic patients eliminate the problems caused by asthenopia by exploring its possible risk factors in the myopic population. Our research focused mainly on analyzing the relationship between eye habits and asthenopia in myopic people.

We found that the most common symptoms that myopic patients experience when performing close work are blurry vision, eye pain or soreness, and feeling tired, which make close work or studying uncomfortable. The prevalence rate of asthenopia is 57% in our study, whereas other studies have reported a prevalence rate of 12.4–32.2% in children below 18 years old, up to 57% in students below 30 years old [5, 29]. A total of 39% of children in our study had asthenopia, and the average age was 12.8 ± 2.6 years old. Age is positively correlated with the incidence of asthenopia in the general population until age 40, at which point the rates decrease [13]. This result is consistent with the prevalence of asthenopia in this study, though our study did not have data for people over 40 years old.

Certain results contradict the effect of gender. Contrary to our results, specific studies have reported a higher prevalence of asthenopia symptoms in women [1, 3, 30], whereas Han et al. and Agrawal et al. found no significant difference in the prevalence of asthenopia between men and women [29, 31], the same as in our study.

Previous research reported that dry eye was positively related to the occurrence of asthenopia [11], and our study also confirmed that dry eye is related to asthenopia in the myopic population. Reduced tear BUT and decreased tear production in dry eye patients may lead to visual fatigue such as shortened near-work time, temporary blurred vision, and eye pain or soreness.

Previous studies have shown that near exophoria and accommodative facilities are related to asthenopia, and asthenopia can be reduced after treatment [7, 25]. However, our research showed a different result: near exophoria and accommodative facilities did not affect the occurrence of asthenopia in myopia patients, consistent with Hashemiet et al.'s study [1]. This contradictory result may be caused by different participants. The participants in our study were myopic patients with or without accommodative dysfunction, whereas participants in other studies were accommodative dysfunction patients with myopia or hyperopia.

Our study found that different ways of receiving eye care education can also lead to a different prevalence of asthenopia. Patients who received eye care education from hospitals and schools were superior to those who received education from the media and those who did not receive eye care education from any source. No statistically significant difference was noted between education from the media and no eye care education. This finding has never been reported before. We speculated that this finding may be due to the pieces of information in the media being mixed with facts and misconceptions so that people could not distinguish between them. These findings show the importance of the sources of eye care education.

A negative correlation between time spent outdoors and asthenopia was observed, though the difference was not statistically significant. Our study showed that asthenopia decreases with an increase in outdoor activity time, but there was no statistical difference. This result may be related to the grouping setting. In future work, we should further divide the group of people with more than 5 hours of outdoor activities to study whether long-term outdoor activities will affect asthenopia or not.

After eliminating collinearity through logistic regression, our study showed that eye care education and continuous near-work time were independent risk factors for asthenopia. Previous studies have shown that continuous near-work time could also affect the progression of myopia [26, 32]. These results suggest that schools and hospitals should strengthen education on eye care for myopic people.

This study also has certain limitations. Our study is a single-center, cross-sectional study with a small sample size. The study population ranged from 9 to 40 years old, with no data for people over 40 years old and did not separate adults and children for comparison. Unlike adults, children are in the developmental stages. Adults of different ages need to be recruited for further study, and further segmentation of those who spent more than 5 hours outdoors should be considered. In the future, we aim to expand the study population and collaborate with researchers from different regions to explore the risk factors for the myopic population with asthenopia and apply our findings to prospective studies.

## 5. Conclusion

This study set out to explore the risk factors of asthenopia in the myopic population. The study finds that receiving eye care education from schools and hospitals and limiting the time of near-work to less than 45 minutes per session could reduce asthenopia. For myopic patients, consciously limiting the time of continuous near work can not only delay the progression of myopia but also reduce asthenopia. In current times, people's inescapable access to electronic products, especially with the rise of online classes and office work, may exacerbate the progression of myopia and asthenopia. Overall, our results demonstrate that eye habits may affect the prevalence of asthenopia in myopic patients. This study's findings may be a more economical and convenient way for myopic people to relieve asthenopia.

## Figures and Tables

**Table 1:** Comparison of eye habits and ocular examination results in asthenopia and non-asthenopia myopia patients.

	Asthenopia	Non-asthenopia	Statistics	*p*
	*n* = 37	*n* = 28	(*Z*, *χ*^2^)	
Age (y)	20 (16, 24)	14.5 (10.25, 20)	*Z* = −2.887	**0.004 ** ^*∗∗*^
Gender, male, *n* (%)	20 (54.1)	15 (53.6)	*χ * ^2^ = 0.001	0.969
Occupation, student, *n* (%)	21 (56.8)	22 (78.6)	*χ * ^2^ = 3.388	0.066
Eye habits				
Weekly outdoor activity time, *n* (%)				
≤2 hours	18 (48.6)	8 (28.6)	*χ * ^2^ = 4.398	0.036
2 to 5 hours	9 (24.3)	5 (17.9)		1.0
>5 hours	10 (27)	15 (53.6)	Reference	
Daily screen time, *n* (%)				
≤5 hours	8 (21.6)	16 (57.1)	*χ * ^2^ = 8.640	**0.003** ^†^
5 to 8 hours	7 (18.9)	4 (14.3)		0.701
>8 hours	22 (59.6)	8 (28.6)	Reference	
Continuous near-work time, >45 min per session, *n* (%)	32 (86.5)	12 (42.9)	*χ * ^2^ = 13.873	**<0.001 ** ^*∗∗∗*^
Where to get eye care education, *n* (%)				
Noneducation	12 (32.4)	2 (7.1)	Reference	
Schools and hospitals	15 (40.5)	25 (89.3)	*χ * ^2^ = 9.643	**0.002** ^†^
Media	10 (27)	1 (3.6)		1.0
Ocular examination				
SE, D	−3.25 (−5.5, −1.0)	−2.75 (−4.0, −1.9)		0.595
Astigmatism, D	−0.5 (−1, 0)	−0.625 (−1.25, 0)		0.128
Anisometropia, *n* (%)	8 (21.6)	5 (17.9)	*χ * ^2^ = 0.141	0.707
Dry eye, *n* (%)	13 (35.1)	2 (7.1)	*χ * ^2^ = 7.035	**0.008 ** ^*∗∗*^

SE means spherical equivalent.  ^*∗∗*^ indicates *p* < 0.01;  ^*∗∗∗*^ indicates *p* < 0.001; and ^†^indicates *p* < 0.017.

**Table 2:** Comparison of near exophoria and accommodative facilities in asthenopia and non-asthenopia myopia patients.

	Asthenopia	Non-asthenopia	Statistics	*p*
	*n* = 27	*n* = 6	(*t*)	
Near exophoria, *n* (%)	13 (48.1)	3 (50)		1.0
Binocular accommodative facility, cpm	7.35 ± 3.06	8.41 ± 1.31	*t* = −0.83	0.414

**Table 3:** Binary logistic regression analysis to assess risk factors of asthenopia.

	OR (95%CI)	*p*
Age		
<18 years old	Reference	
≥18 years old	0.503 (0.101, 2.5)	0.401

Continuous near-work time		
≤45 min	Reference	
>45 min	4.227 (1.027, 17.39)	**0.046**

Time spent on electronic devices per day		
≤5 hours	Reference	
>5 hours	2.619 (0.604, 11.347)	0.198

Where to get eye care education		
Noneducation or media	Reference	
Schools and hospitals	0.115 (0.024, 0.541)	**0.006**

Dry eye		
Nondry eye	Reference	
dry eye	3.978 (0.506, 31.296)	0.190

CI means confidence interval.

## Data Availability

The data used to support the findings of this study are included within the article.

## Supplementary Materials

Supplementary Materials The questionnaire of asthenopia symptoms we used in the study was provided in supplementary files.
