# Supplementary material for: Eye Habits Affect the Prevalence of Asthenopia in Patients with Myopia
Source: J Ophthalmol. 2022 Oct 17;2022:8669217. doi: 10.1155/2022/8669217 (PMC9592227; doi:10.1155/2022/8669217)
Supplement: Supplementary Materials — The questionnaire of asthenopia symptoms we used in the study was provided in supplementary files. [file 8669217.f1.docx]

**Annexure 1: Questionnaire of asthenopia symptoms**

|  | Never | Infrequently | Sometimes | Fairly Often | Always |
| --- | --- | --- | --- | --- | --- |
| score | 0 | 1 | 2 | 3 | 4 |
| 1. Do your eyes feel tired or uncomfortable when reading or doing close work? | | | | | |
| 1. Do you have headaches when reading or doing close work? | | | | | |
| 1. Do you feel sleepy when reading or doing close work? | | | | | |
| 1. Do you lose concentration when reading or doing close work? | | | | | |
| 1. Do you have trouble remembering what you have read? | | | | | |
| 1. Do you have double vision when reading or doing close work? | | | | | |
| 1. Do you see the words move, jump, swim, or float on the page when reading or doing close work? | | | | | |
| 1. Do you feel like you read slowly? | | | | | |
| 1. Do your eyes ever hurt or sore when reading or doing close work? | | | | | |
| 1. Do you feel a “pulling” feeling around your eyes when reading or doing close work? | | | | | |
| 1. Do you notice the words blurring or coming in and out of focus when reading or doing close work? | | | | | |
| 1. Do you lose your place while reading or doing close work? | | | | | |
| 1. Do you have to re-read the same line of words when reading? | | | | | |
| 1. Do you avoid close reading or doing close work? | | | | | |
| 1. Do you feel difficult to focus when looking from a distance back up to close reading? | | | | | |
